# An Open Letter

**Published:** 1893-03

**Authors:** J. R. Haynes

**Affiliations:** 264 N. Illinois Street, Indianapolis, Ind.


### AN OPEN LETTER.

### "Dr. Wesselhœft Throws Down the Gauntlet."

There appears in the *Homœopathic Recorder* for November, 1892, p. 254, a letter from Dr. Conrad Wesselhœft to the publishers, in which he takes great umbrage at what I said in a paper to the I. H. A., "Is there a law of similars?" published in The Homœopathic Physician, p. 448, of last year. In this paper I said that I could not recommend Dr. Conrad Wesselhœft's translation of Hahnemann's *Organon.*

It seems to me that I have read somewhere, or that I have heard some one repeat, that a certain reformer on a certain occasion said to those around him, "Where much is given, much would be required; but where little was given, little would be required."

As I do not live in the "Hub," and stand upon the great pedestal of knowledge around which the whole universe revolves several thousand times each year, but in a little town on one of the great Western plains, it seems to me that it would not be asking too much if I claimed protection under the second clause of the above.

I will state that I wrote out the circular, sent it to the printer, that in due time a proof was returned, which I corrected and sent back, then devoted my spare time to the directing of envelopes; and when the circulars came in my folks folded them and sent them away as fast as possible. I did not examine the circular, supposing that it was correct, until my attention was called to it by one to whom it had been sent, when I found that my corrections had not been made, but others introduced which were much worse.

The circulars had been sent all over the country, and it would be impossible to recall them, and as I had nothing whatever to do with the printing or the miscorrections of the circular, I let it pass for what it was worth; this is all the explanation I have to make regarding it. I am quite sure that I mailed one of them to Dr. Conrad Wesselhœft at the time, and will say that he need not go to so great expense for its preservation as I will send him one at any time if he will send me one cent for postage.

If I said anything about his "translation" being a failure *as to sales*, I had no intention of doing so, as I knew nothing about how many of them had been sold, but in a foot-note the publishers say "For the year ending October 1st, 1882, the sale of *The Organon* amounted to five hundred and twenty-three copies. Of these half were sold through Bœricke & Tafel, Chicago."

But they do not tell us how many copies have been sold up to October 1st, 1892. I am well aware that two colleges have demanded that their students should supply themselves with copies of *The Organon*, and as there were no other edition available they were *compelled* to purchase that one. Now is there any more evidence required than *their own* to prove that it was a failure as to sale?

Now if I can figure it out correctly, this would be one copy to a little over four hundred of those who are claiming to practice homœopathically. To make it go around they would have to serve it as "the old fellow" did his Bible: give each a part of a leaf.

My opinion was formed as to its failure largely by the value which was placed upon it by those physicians who had purchased it, many of whom were in possession of the old "*Allentown Translation*," which they prized so highly that they kept it under lock and key, whilst they offered to sell "my translation" for the munificent sum of fifty cents, as they had no further use for it.

I am well aware that there are some errors in the old edition, "But with all thy errors, I love thee still." Let me ask, why does Dr. Conrad Wesselhœft, if he made an independent translation, *copy* those errors?

It has been my private impression for a long time that Dr. C. Wesselhœft *did not* make a translation of *The Organon*, but *copied the old editions* as far *as it suited him*, and garbled the rest, so that neither he nor any one else could make anything out of it, and it is not my belief alone. Perhaps we are "deficient in intelligence or education, or both."

"I accepted the duty of translator entirely at Dr. Hering's most urgent request, and never heard a word of dissatisfaction during the two years in which Dr. Hering had my manuscript in his hands, regarding which we corresponded freely in the most friendly manner."

All that I have to say upon that subject is, that Dr. Hering told me in his own office that he was not an English scholar, and that he had no time to perfect himself in the English language, and that he transacted his business and did his writing in the German language, which was good enough for him.

"When it was finally published by your firm, my translation also received Dr. Dunham's unqualified indorsement, as well as that of others whose opinions are weighty."

As he does not tell us "whose opinions are weighty," we shall have to pass them over for what they are worth.

If my memory serves me correctly, Dr. Dunham died the morning of February 18th, 1877, some several years before "my translation" was published, and if Dr. Dunham gave it his "unqualified indorsement" he must have done so through some of those spirit mediums which were so plenty in the "Hub," and if so he must have changed his views most remarkably from what they were when I had the honor of his acquaintance.

"The present enthusiastic praise of the old edition is absurd in the face of its former condemnation by the purest of the pure."

Will you please tell us to whom you allude as "the purest of the pure"? Then perhaps we may be able to form our own opinion.

"To say that if my translation is correct then all others he has seen are certainly incorrect is proof that he is incapable of understanding any of them, as they all say the same thing, each in its own manner of expression."

This is just what we do not want, we want the "expression" just as Hahnemann gave it.

"This will be found to agree perfectly with the German text, of which my critic is entirely unable to judge, and is guilty of an intentional insult unless he can point out my errors. This I challenge him to do."

Perhaps he (Dr. Conrad Wesselhœft) has forgotten what the late Dr. Ad. Lippe said, viz.: "The mistranslation of Conrad Wesselhœft, in which to find a shadow of Hahnemann's teachings would require a better microscope than has been invented." (See HOMŒOPATHIC PHYSICIAN, Vol. I, p. 139, and the allusions in other volumes concerning it.)

To point out his errors would be to rewrite the whole *Organon*, which I have not time to do. When any one translates another's writings and foists them upon the public as the original, he should do so as perfectly as possible or else he has no right to call it a translation. This Dr. Conrad Wesselhœft has not done, nor can he make others believe that he has done so.

"There are some that make capital out of the pretense that they possess a key to the mystery, which is discussed and still more profoundly mystified in their *Organon* meetings. Dr.

Haynes is a first-rate example of those who have been hopelessly gulled by such influences."

He was never "gulled" by "my translation," for he put it down as a first-class humbug after its first reading, and has seen no reason to change that belief up to the present time, and he is not alone in thinking of it in the same way. There may be others who are "still more profoundly mystified," but I have never heard of them until told so by Dr. Conrad Wesselhœft.

"To those who have really read it, *The Organon* is one of the best written and logical books in the world."

I will admit that as true, providing they have "it" as it was promulgated by Hahnemann himself, and will go a little further, and say that its equal was never written by man, and that the person does not exist, and never will, that can practice true Homœopathy who has not first made himself familiar with *The Organon* as Hahnemann wrote it; hence, the eclecticism that is being palmed off as Homœopathy.

"Without such qualifications the reader wastes his time, which he might employ more profitably in the hay-field or the shop. So much for intelligence and intelligibility."

If Dr. Conrad Wesselhœft will place his communication in the hands of some little school girl, who is attending her first term in the primary department of any of our common schools, she will very soon point out to him who has the best title to the hay-field or the shop.

"Drs. Stratton, Dudgeon, and I have done the best we could, and I am sure that both of the other translators would agree with me that if a member of the I. H. A., or any other man, is unable to understand our translations, the fault lies in his deficient intelligence or education or both."

This may be true, but as we were not all educated in the "Hub" we must be allowed to see things somewhat differently, and especially upon the subject of "my translation" of what he calls Hahnemann's *Organon.*

If the whole profession were so profoundly satisfied with "my translation," why did the American Institute and Congress pass an *unanimous* resolution asking Dr. Dudgeon to make *another*

translation of *The Organon* together with notes and comments? Was it simply done for buncombe? Did they intend to throw off on him as soon as it was done or was it done in good faith?

Another very serious objection to "my translation:" When Hahnemann wrote his different editions of *The Organon* he added notes, and more especially in his fifth edition. These he placed *under the sections to which they belonged*, and called attention to them in the text as he passed along. These notes were his latest thoughts up to the time of its issue; many of them are of great importance as much or more so than the text itself; for they *explain the text.*

In "my translation" this has not been done; they are placed at the *back* of the book *where they will not be seen*, and no attention *is called to them in the text;* they are jumbled up—*e. g.*, (2) 6; (10) 22; (150) 289, etc., all through them.

Sections 293 and 294 are placed at the extreme end of the volume. In these Hahnemann called particular attention to the subject of Mesmerism, telling his readers that without great care in attempting to use such a powerful influence they were liable to do great mischief and cause an irreparable wrong.

*Was this as Hahnemann intended it should be done?*

I am willing to admit that it is not the easiest thing in the world "to find out just what Hahnemann has said," and I would be very much pleased if, by fair means, I could get possession of the *unpublished papers which were left by him.*

I think that I am very well supplied with different copies of *The Organon.* I have the second, third, and fifth editions, and the Lutze editions in German, the old Allentown translation, the Stratton translation, the pretended Conrad Wesselhœft "translation," and all that has been published of Dr. Fincke's translation (up to section 284) published in the *Journal of Homœopathics* by Dr. Hitchcock, and should Dr. Dudgeon make another translation I shall endeavor to procure a copy of that also.

In early life I inherited a pair of German shoes (ein paar Holzschuhe), and I found by wearing them awhile each day that after a time I could spell out a few words of the German language, and by diligently wearing them after a considerable time I could translate some of the sections of Hahnemann's *Organon* that were at least satisfactory to *me*, but to satisfy myself still further I procured the services of a German (who had spent twenty years teaching in the colleges at Heidelburg and some fifteen years teaching in this city what were called the higher branches of the German language) to make translations of a number of the sections from the Fifth Edition of *The Organon;* these I compared with the sections that were translated by Dr. Fincke. I found them to agree in every particular, and perhaps I could have done so with the Dr. Conrad Wesselhœft's "translation" if I had a pair of spectacles that had been made in *Boston;* but I could not without them. By these comparisons, and those that I have been able to make, I can but think that Dr. Fincke has made as honest and true translation as is possible.

These are my reasons for wishing to see Dr. Fincke's translation published, as well for my own use, as to have a perfectly reliable translation that we can put into the hands of our students from which they can learn how to practice true Homœopathy.

I would recommend that every homœopathic physician supply himself with a copy of *The Organon* in the German language, and by some little study and comparisons with different translations, he will soon be surprised to see how *The Organon* will explain itself to him. And I will say to Dr. Conrad Wesselhœft: Come on mit your "Corrigenda."

Very respectfully yours,

J. R. Haynes.

264 N. Illinois Street, Indianapolis, Ind.

